# Systemic Sodium Thiosulfate as an Adjunct Treatment in Calcinosis: A Retrospective Study

**DOI:** 10.3390/jcm12247741

**Published:** 2023-12-17

**Authors:** Lili Róbert, András Bánvölgyi, Kende Lőrincz, Péter Holló, Bernadett Hidvégi

**Affiliations:** Department of Dermatology, Venereology and Dermatooncology, Faculty of Medicine, Semmelweis University, 1085 Budapest, Hungary; robert.lili@med.semmelweis-univ.hu (L.R.);

**Keywords:** intravenous sodium thiosulfate, oral sodium thiosulfate, dystrophic calcinosis, calcinosis cutis, calcinosis therapy

## Abstract

(1) Background: Calcinosis of the skin mainly appears in connective tissue disorders (dystrophic subtype). It may cause inflammation, ulceration, pain, and restricted joint mobility. Management is difficult; sodium thiosulfate is one potential therapeutic agent with promising data on intralesional and topical formulation for smaller calcified lesions. There are very limited data on systemic administration. (2) Methods: A retrospective study was conducted at our department to assess the efficacy of oral and intravenous sodium thiosulfate in dystrophic calcinosis between 2003 and 2023. (3) Results: Seven patients were identified, who received systemic sodium thiosulfate (intravenous or oral). The mean duration of calcinosis at the time of administration was 3.8 ± 4 years (range 0–11). Intravenous sodium thiosulfate was administered in doses of 12.5–25 g two or three times during one week of the month for 4.5 ± 3.9 months on average. Orally, 1–8 g was administered daily for 29.1 ± 40.9 months on average. Four of seven patients had a partial response (57.1%). Despite no complete response, pain, ulceration and inflammation frequency decreased, and sodium thiosulfate prevented further progression in responsive patients. (4) Conclusions: Based on our experience and literature data, systemic sodium thiosulfate may be a potential adjunct therapy in calcinosis, especially if inflamed or ulcerating.

## 1. Introduction

Calcinosis cutis is an ectopic deposition of calcium salts that occurs most commonly in autoimmune connective tissue diseases (ACTDs) or in soft tissue damage in other disorders in the absence of altered calcium and phosphate levels (dystrophic subtype) [1,2,3]. It may lead to complications such as joint movement restriction, pain, or ulceration. Treatment is so far unresolved, and recommendations are based on case reports and small case series.

Therapeutic options include pharmacotherapy (calcium channel blockers [4,5], bisphosphonates [5,6,7], probenecid [5,8,9], aluminum hydroxide [5,10], warfarin [11], ceftriaxone [12], intravenous immunoglobulin [13,14,15], colchicine [4,16], minocycline [5,17], thalidomide [18], intralesional corticosteroids [19,20], and rituximab [21,22]), surgical excision [2,4,5,23], carbon dioxide laser [24], and extracorporeal shock wave lithotripsy [25,26].

Sodium thiosulfate (STS) is a potential agent with antioxidant, chelating, and vasodilating effects, although it remains unclear how it dissolves calcification [27,28,29]. It may be related to triggering the production of mineralization inhibitors, preventing precipitation, restoring endothelial function, and improving blood supply [30,31,32,33,34]. A favorable effect has been demonstrated in small lesions given intradermally or topically [35,36], and it has also shown efficacy in another calcifying disorder, calciphylaxis with systemic administration [37,38,39]. These positive results support the systemic use of this agent in multilocated calcinosis where intralesional administration is impractical.

We observed an improvement in inflammation and ulcerative tendency in some of the calcinosis patients treated with systemic STS. The aim of this study was to collect data on the efficacy and to determine its place and relevance in the management of calcinosis. Given the paucity of data in the literature, and the difficulties of research due to the marked heterogeneity of this scarcely investigated disorder, observational studies are of paramount importance as a source of information. To the best of our knowledge, our paper is the first to report higher doses and to summarize the current knowledge.

## 2. Materials and Methods

In this retrospective observational study, we aimed to collect data on the efficacy of systemic (oral and intravenous) STS therapy in patients with dystrophic calcinosis from a single center.

We retrieved cases coded as calcinosis cutis according to the International Classification of Diseases from our medical report system in our department of dermatology between January 2003 and January 2023. This was followed by a manual search for cases of dystrophic calcinosis in which intravenous or oral STS was administered. Seven cases were identified with this method. The clinical, radiological, laboratory and photographic records of these patients were evaluated. The following data were collected: sex, underlying disease, activity of underlying autoimmune connective tissue disease, prior and concomitant immunosuppressive therapy, localization of calcinosis, extent of calcification, (estimated) number of calcified lesions, presence of ulceration, age at onset of calcinosis, age at the start of STS therapy, duration of calcinosis at start of STS therapy, dosage and frequency of intravenous and oral STS therapy, duration of treatment, response, method of evaluation of the response, serum calcium and phosphate levels before, during, and after treatment, prior and concomitant therapy against calcinosis, side effects, patient complaints, patient compliance, and reason for discontinuation of STS therapy.

Oral STS was administered in intestine-soluble capsules prepared in our university pharmacy. The capsules contained 1–2 g of sodium thiosulfate pentahydrate (Merck KGaA, Darmstadt, Germany) and no excipients. For intravenous treatment, one or two STS ampoules (12.5 g STS/ampoule) were diluted in 500 mL saline and infused over 30 min. The STS solution contained the following ingredients per liter: 392.3 g of sodium thiosulfate pentahydrate (Merck KGaA, Darmstadt, Germany), 5.22 g of disodium phosphate dehydrate, sodium hydroxide if necessary for pH adjustment (maximum of 0.001 g), and water for injection up to one liter. The dose and duration of treatment varied according to side effect tolerance and the clinical scenario. Serum calcium and phosphate levels remained within normal ranges before and after treatment. Response was considered a complete response in the case of total healing. Partial response was defined as a reduction in lesion size, a reduction in the number of surgical procedures needed, a reduction in pain, an improvement in the mobility of the adjacent joints, or stabilization with treatment and progression after discontinuation. No response was defined as no change in lesion size, number, and associated complaints or progression. For the outcome evaluation, we used clinical reports of the treating physician’s assessment, documented patient complaints, radiological reports of radiography, ultrasound, computed tomography or magnetic resonance imaging examinations, and photographic documentation during treatment.

Due to the retrospective observational nature of the study, the results are presented using descriptive methods, means, ranges, and time intervals. The method does not allow for the provision of statistical analysis in the absence of standardized follow-up and evaluation.

## 3. Results

Data on demography, characteristics of calcinosis, STS dosage, administration frequency, and therapeutic response are summarized in Table 1.

### 3.1. Patient’s Characteristics

Male to female ratio was 1:6. The mean age at onset of calcinosis was 41.1 ± 15.6 years (range 25–70). The mean duration of calcinosis at the time of STS administration was 3.8 ± 4 years (range 0–11).

### 3.2. Underlying Disease

All the patients had dystrophic calcinosis complicating an ACTD. Underlying autoimmune diseases were systemic sclerosis (n = 2), systemic lupus erythematosus (n = 2), dermatomyositis (n = 1), mixed connective tissue disease (n = 1), and localized scleroderma (morphea) (n = 1). During treatment, all of the patients were in relatively good general health except for inflammation of the calcinosis lesions. During STS treatment, six of seven patients were in an inactive phase of the underlying autoimmune disease, and only one patient had progressive systemic sclerosis. Four of seven patients were receiving concomitant immunosuppressive therapy for the underlying ACTD. Oral methylprednisolone was most commonly administered in low doses in three of seven patients. Three of seven patients did not receive concomitant immunosuppression during STS therapy. Prior or concomitant immunosuppressive therapy failed to resolve calcinosis lesions or reduce inflammation.

Three of seven patients received prior treatment for calcinosis, including diltiazem, colchicine, doxycycline, and extracorporeal shock wave lithotripsy. None of these therapies proved to be effective on the lesions. Only one patient received additional treatment for calcinosis with diltiazem. In this case, diltiazem had been administered prior to STS without any effect and was continued in cardiological indication after the addition of STS.

In one case, the treatment was complicated by secondary hyperparathyroidism, which occurred years after the onset of dystrophic calcinosis of autoimmune origin (mixed connective tissue disease). Hyperparathyroidism causes alterations in the calcium and phosphate homeostasis that can lead to calcification by a so-called metastatic mechanism, and this imbalance in the calcium-to-phosphate ratio maintains a calcification-promoting microenvironment.

### 3.3. Characteristics of Calcinosis

The localization of the calcinosis was the lower extremities (mainly shins) in two of seven patients, the trunk in one patient, and widespread in one patient. The most common localization was the upper extremities mainly around the joints (elbows, wrists, fingers) in three of seven patients. Most of the patients had multiple calcinosis lesions, except for two patients. One of them had two larger ulcers, and the other one had four calcified lesions. The estimated extent of calcinosis (percentage of total body surface area (TBSA%)) ranged from 0.25% to 4% (mean 2.2%). Five of seven patients had ulceration at the calcinosis site during STS treatment.

### 3.4. Subjective Complaints of the Patients

Patient complaints included pain in four of seven patients, restricted joint mobility in three of seven patients, and frequent inflammation of the nodules in three of seven patients. The most common complaint was ulceration of the nodules and discharge of a purulent or chalky substance from the nodules or ulcers in five of seven patients.

### 3.5. Characteristics of STS Therapy

Five of seven patients received STS via intravenous route followed by oral administration, one patient received intravenous and oral STS simultaneously, and one patient received oral STS only.

Intravenous STS was administered in doses of 12.5–25 g two or three times during one week of the month. Total doses ranged from 50 to 125 g/month. The duration of treatment ranged from one to twelve months (mean 4.5 ± 3.9 months).

Oral STS was administered in doses of 1–8 g daily for 7–119 months (mean 29.1 ± 40.9 months).We used higher oral doses in general than were reported in the literature. The decision to switch from intravenous to oral administration was based on patient convenience or better tolerability of side effects.

Two of seven patients are still on oral STS therapy with stable disease and no side effects. In the other five cases, the reason for the discontinuation of STS therapy was the clinician’s decision due to the lack of therapeutic response in two of seven patients, patient’s decision due to difficulty in access due to remoteness of residence in one in seven, loss to follow-up in one in seven, and sudden death from external causes in one in seven.

Adherence to STS therapy was good in five of seven patients. Two patients were non-compliant.

### 3.6. Side Effects

Gastrointestinal side effects (nausea, diarrhea) were frequent but mostly tolerable in five of seven patients. Nausea complicated intravenous administration, which could be prevented in most cases by intravenous metoclopramide given shortly before the STS infusion. Mild diarrhea complicated oral therapy in two of seven patients. Apart from fatigue, there were no other significant side effects. No persistent or severe side effects were observed.

One patient had osteoporosis prior to treatment with STS. As STS is a chelating agent, we were concerned about the progression of osteoporosis. The patient received calcium supplementation during the STS treatment. Regular osteodensitometry showed no progression and no significant risk of fracture. Despite calcium supplementation, STS was able to achieve a therapeutic effect. Follow-up of this patient for 119 months without any relevant side effects suggests the safety of STS.

### 3.7. Outcome

**Figure 1:**
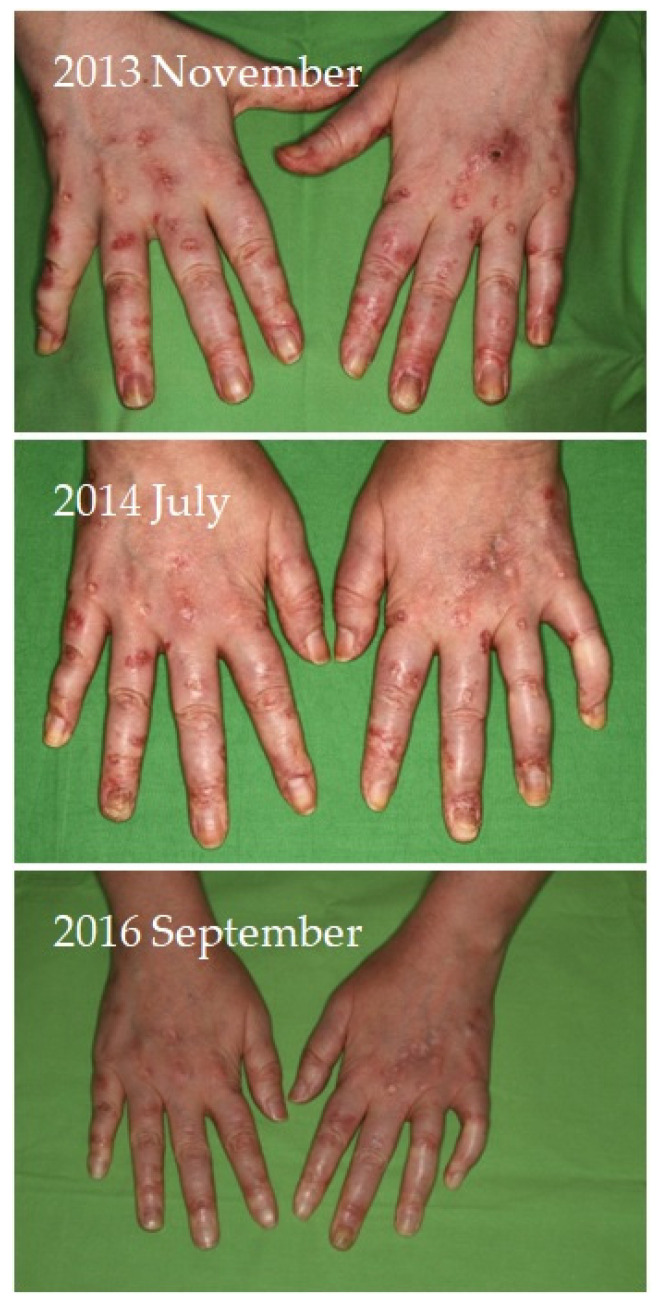
Radiologically confirmed calcified lesions of the hands of a female patient with systemic lupus erythematosus showing continuous regression during administration of systemic sodium thiosulfate photographed at initiation, 8 months, and 3 years of treatment. The patient is still receiving oral sodium thiosulfate therapy with calcinosis lesions being stable.

**Figure 2:**
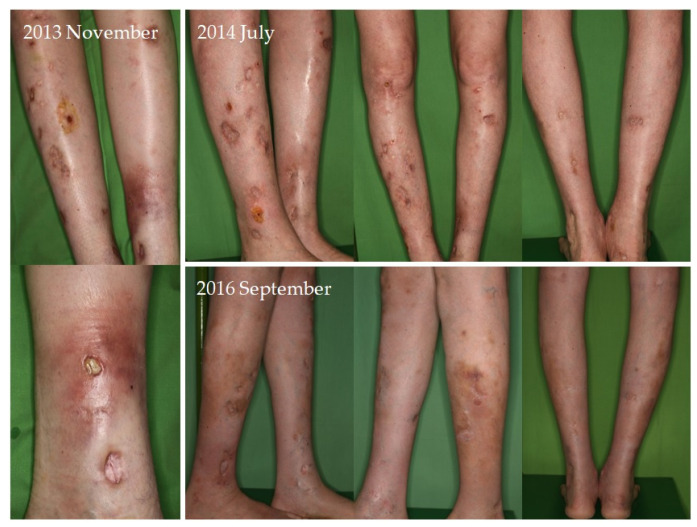
Radiologically proven ulcerated calcinosis lesions of the lower extremities in a female patient with systemic lupus erythematosus showing improvement with significantly less frequent infections during administration of systemic sodium thiosulfate. Photographed at initiation, 8 months, and 3 years of treatment. The patient is still receiving oral sodium thiosulfate therapy with calcinosis lesions being stable.

**Figure 3:**
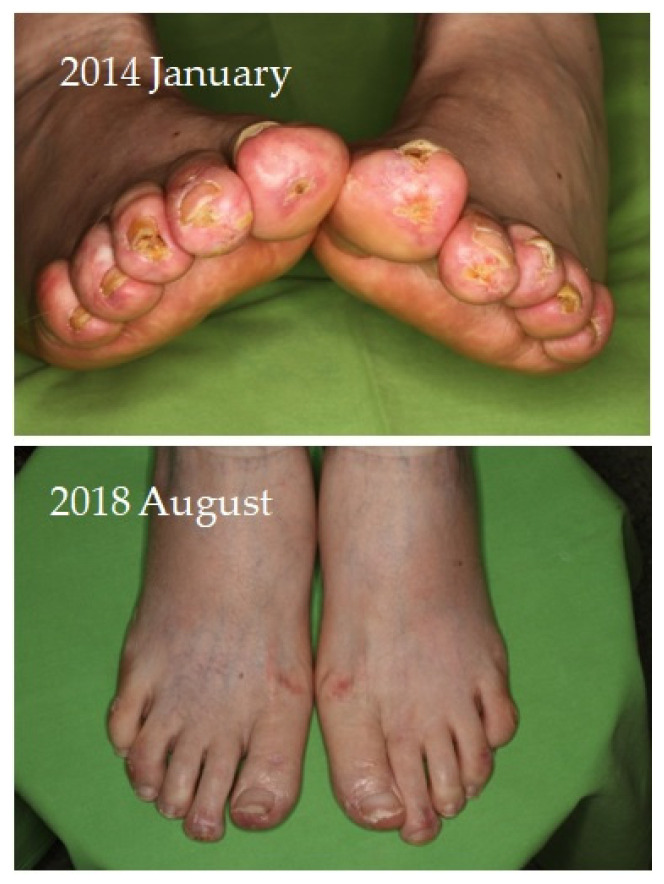
Improvement of the calcified lesions on the feet of a female patient with calcinosis cutis receiving sodium thiosulfate therapy photographed after 2 months of initiation and 4 years of treatment.

**Figure 4:**
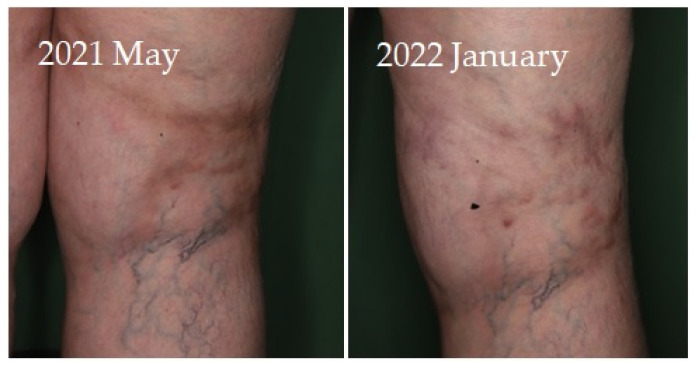
Improvement of the inflammation and stabilization of calcified lesions on the thigh of a female patient with dermatomyositis receiving sodium thiosulfate therapy photographed at initiation and at 6 months of treatment. Patient reported cessation of pain after the first three STS infusions.

Four of seven patients had a partial response (57.1%) to systemic therapy and three had no response. No complete response was observed. Partial response was defined as clinical improvement, reduction in number and/or size of the calcified nodules or ulcers, softening of the nodules, cessation of pain, reduced frequency of inflammation, reduced need for surgical intervention due to inflamed suppurative lesions, improved mobility of the adjacent joints, stabilization of the lesions, or progression after discontinuation. Improvement was seen mainly in smaller inflamed or smaller inflamed and ulcerated lesions (Figure 1, Figure 2, Figure 3 and Figure 4). Partial responses were observed mainly in patients who were able to take 8 g STS orally daily, mainly as a reduction in symptoms and complications. In patients who could only tolerate lower doses due to gastrointestinal side effects, no response was observed. In some of the patients, although the improvement was not confirmed radiologically, patients reported the cessation of pain, increased mobility, and less frequent inflammation.

## 4. Discussion

Calcinosis cutis is a poorly studied condition and its pathophysiology is not well understood, although it affects many patients suffering from a wide range of connective tissue disorders. The largest group is calcinosis in autoimmune connective tissue diseases. The most critical problem is inflammatory and ulcerative calcinosis with associated pain and risk of wound infection, particularly in these patients who are usually not in a satisfactory general health condition. Autoimmune disease is often associated with impaired peripheral blood supply to the soft tissues and reduced immune defense. Therapy-related immunosuppression increases susceptibility to infections. As a result, ulcerative calcinosis can have serious consequences in the context of inherently impaired wound healing and reduced immune function, not to mention the complicating effects on daily life, as calcinosis tends to form in the acral areas most exposed to microtrauma (e.g., fingertips, elbows, knees). This not only makes life miserable for patients but also has a negative impact on the healthcare system and the economy. Inflamed nodules often require surgical intervention, and frequent superinfections require antibiotic therapy. Reduced mobility and pain associated with the lesions may leave patients unable to work or even do household chores, which is a particular problem as the population affected is relatively young. Ulcerative calcified lesions are hard to heal because the calcified deposits act as foreign bodies that can interfere with the healing process. Therefore, the removal of the crystal deposits is desirable. As tissue alterations are maintained in most of the underlying diseases of dystrophic calcinosis, new lesions are expected to form after surgical excision. Surgical removal of the calcified lesions is most appropriate in cases where the causative agent is not persistent or where the underlying disease can be cured by the surgery itself, such as in calcifying neoplasms. Deposits in calcinotic nodules or plaques may lead to ulceration of the overlying skin due to increased pressure and impaired microcirculation, thus STS should also be considered in the prevention of ulceration in calcinosis.

In the literature, only ten cases of dystrophic calcinosis treated with systemic STS have been reported, which are summarized in Table 2 [28,34,40,41,42]. The frequency and dose of intravenous STS was variable, with 25–75 g of STS administered weekly. Systemic STS (intravenous and combined intravenous–oral therapy) relieved the discomfort associated with calcinosis and prevented further progression in five of ten cases. It should be noted that one patient was also receiving concomitant abatacept therapy, which may have influenced the outcome. Inflammation of the calcified nodules requiring surgical intervention was also quite common, and this tendency was also alleviated by systemic STS, although a complete response was not achieved.

Half of our patients also benefited from STS, despite no complete resolution, which is consistent with the literature data suggesting that approximately half of patients respond to this treatment. STS therapy resulted in stable disease. Therapeutic achievements diminished after discontinuation, suggesting that STS may be best used in a continuous manner. It also reduced the number of surgical procedures required for painful, inflamed, infected calcinosis nodules [42]. Overall, it was mainly effective in preventing progression and inflammation, relieving associated symptoms, and improving ulcers. Based on our experience and the literature data, the potential role of oral or intravenous STS as an adjunctive therapy in the management of calcinosis associated with ACTD should be re-evaluated, particularly in unstable, progressive lesions and inflammatory ulcerative calcinosis. In these cases, STS may reduce inflammation, which could lead to ulceration and aid wound healing through its vasodilator effect. Systemic STS therapy appears to be safe [42], as suggested by our case where STS was administered for more than 10 years without any significant side effects. Oral administration has fewer and more tolerable side effects compared to intravenous administration (diarrhea vs. vomiting, metabolic acidosis, dizziness, and fatigue), and it also lacks the risk of injection site infection. Hypothetically, distributed dosing may result in maintained plasma concentrations, which may be beneficial for these patients.

The limitations of this study are its retrospective observational nature, the small size of the population, the different dosing regimens, and the qualitative assessment of the results by clinicians. Therefore, no far-reaching conclusions can be drawn. Nevertheless, to the best of our knowledge, only very few case reports and two case series of three and four patients have been published considering systemic STS therapy, especially oral STS in dystrophic calcinosis, and our paper is the first one to summarize the current knowledge. In addition, we used significantly higher doses of oral STS compared to the literature cases.

Calcinosis is a rare complication in several different background diseases, making it very difficult to establish comparable groups due to heterogeneity. The activity of the underlying autoimmune diseases, which requires additional immunosuppressive therapy also has a strong influence on the clinical course, so that potential therapeutic agents cannot be tested in the same clinical scenario in different patients. This makes randomized controlled studies almost unfeasible. On the other hand, the management of calcinosis is still an unresolved issue, with no evidence-based treatment guidelines and insufficient clinical experience to guide clinicians in the management of patients. For this reason, observational studies are of paramount importance in the management of calcinosis, as they are currently one of the few sources of information available. We hope that our observations will contribute to this body of knowledge. Oral STS at higher doses seems to improve the condition and quality of life in selected patients without significant adverse effects.

## 5. Conclusions

In conclusion, our observations and the literature data suggest that STS, intravenously, orally, combined, or sequential, could be a therapeutic option in calcinosis cutis, or in the prevention of inflammation and ulceration of calcified nodules. It is quite effective in the treatment of calciphylaxis, where the blood supply is impaired, suggesting that it should be considered for those calcified lesions where the blood supply to the affected area is also reduced. Also, in dialysis patients with calciphylaxis, slow elimination contributes to the effect, suggesting that a longer sustained plasma concentration would be desirable. Intralesional STS also appears to be effective in the treatment of smaller calcinosis lesions; therefore, this drug should be studied in different clinical scenarios, such as in less widespread disease and earlier stages. Presumably, it will not be indicated as first-line therapy but may have a role as an adjunct, particularly in inflamed or ulcerated calcinosis, in those patients who can tolerate higher doses, and in disabling calcinosis, which is not curable according to the current knowledge. It may also help reduce the economic burden of this disease and improve patients’ quality of life. Further studies are needed concerning pharmacokinetics, dosage, efficacy, responsive lesion size, and indication. Stabilization of the capsules with extended release may also be a target for investigation. In conclusion, based on the literature and our own data, different formulations of STS may have a potential adjunctive role in the therapy of calcinosis.

## Figures and Tables

**Table 1:** Data on patients treated with systemic sodium thiosulfate for dystrophic calcinosis cutis at our department.

Sex	Under- Lying Disease (Activity)	Prior Immuno- Suppression	Concomitant Immuno- Suppression	Extent of Calcinosis (TBSA%)	Age at Onset of Calcinosis (Years)	Age at Start of STS (Years)	Intravenous STS (g)	Frequency	Duration (Months)	Response	Oral STS (g/d)	Duration (Months)	Response	Side Effect
F	SSc (i)	AZT	-	1	32	38	12.5–25	2–3 x/w/m	2	NR	2–6	9	NR	GI
F	DM (i)	CS, AZT	CS, AZT	3	53	54	12.5–25	3 x/w/m	4	PR	8	36+	PR	none
F	MCTD (i)	CS, MTX, MMF	-	3	25	36	25	3 x/w/m	5	NR	2–4	7	NR	GI
F	SLE (i)	CS, MTX, AZT, CyA	CS	3	36	37	25	4–5 x/m	12	PR	1–8	119+	PR **	GI
F	SSc (p)	CS, MTX, AZT, MMF, CyF, RTX	MMF	1	42	44	25	3 x/w/m	1	PR	2–8	11	PR	none
F	SLE (i)	CS, AZT, chloroquine	CS, AZT	4	70	70	25	2 x/w/m	3	NR	3–4	14	NR	GI
M	deep morphea (i)		-	0.25	30	36	-	-	-	-	2–6	8	PR	GI

Abbreviations: AZT azathioprine, CS corticosteroid methylprednisolone, CyA cyclosporine, CyF cyclophosphamide, DM dermatomyositis, F female, g gram, g/d grams daily, GI gastrointestinal, i inactive, M male, MCTD mixed connective tissue disease, MMF mycophenolate mofetil, MTX methotrexate, NR no response, p progressing, PR partial response, SLE systemic lupus erythematosus, SSc systemic sclerosis, STS sodium thiosulfate, TBSA% total body surface area %, x/m times monthly, x/w/m times per week monthly once, + ongoing at the time of publication, ** concomitant diltiazem therapy

**Table 2:** Data from literature cases treated with systemic sodium thiosulfate for dystrophic calcinosis cutis.

Sex	Underlying Disease	Localization	Ulceration	Age at Onset (y)	Age at Start of STS (y)	Intravenous STS (g)	Frequency	Duration (m)	Response	Oral STS (g/d)	Duration (m)	Response	Side Effect	Author
F	DM	W	Y	11	14	10, then 15	3 x/w, then 2 x/w	0.5 + 3	PR *	-	-	-	none	Arabshahi et al., 2012 [28]
F	MCTD (SLE/DM)	W	N	44	54	12.5	3 x/w	8	PR	-	-	-	none	Badawi et al., 2020 [40]
F	MCTD (SLE/DM)	LE, UE, T	Y	45	50	25	2 x/w	6	NR	-	-	-	GI, fatigue	Song et al., 2018 [41]
F	DM	LE	Y	40	50	25	3 x/w	0.25	NR	-	-	-	GI, fatigue	Song et al., 2018 [41]
F	DM	LE, UE, T, FN	Y	10	20	25	1 x/w	6	NR	-	-	-	GI, fatigue	Song et al., 2018 [41]
M	PXE	W	N	ND	11	25, then 10	3 x/w, then /d	6	PR	-	-	-	GI, fatigue, metabolic acidosis	Omarjee et al., 2020 [34]
F	SSc	LE, UE	Y	60	74	20	5 x/w/m	6	NR	-	-	-	none	Mageau et al., 2017 [42]
F	DM	LE, UE	Y	40	43	20	5 x/w/m, then /2d	6 + 6	PR	1.5	6	PR	catheter infection	Mageau et al., 2017 [42]
F	SLE	LE	N	ND	33	20	5 x/w/m, then 5 x/w/6w	6+	PR	2	6+	PR	GI	Mageau et al., 2017 [42]
M	DM	W	N	9	11	9–17	/d	5	NR	3	1	NR	GI, catheter infection	Mageau et al., 2017 [42]

Abbreviations: DM dermatomyositis, F female, FN face and neck, g gram, g/d grams daily, GI gastrointestinal, LE lower extremity, m months, M male, MCTD mixed connective tissue disease, N no, ND no data, NR no response, PR partial response, PXE pseudoxanthoma elasticum, SLE systemic lupus erythematosus, SSc systemic sclerosis, STS sodium thiosulfate, T trunk, UE upper extremity, W widespread, y years, Y yes, x/w times per week, x/w/m times per week monthly once, x/w/6w times per week once in 6 weeks, + ongoing at the time of publication, /2d every second day, * concomitant abatacept therapy.

## Data Availability

The data that support the findings of this study are available on request from the corresponding author. The data are not publicly available due to privacy or ethical restrictions.
